# Hazard Classification and Stability Analysis of High and Steep Slopes from Underground to Open-Pit Mining

**DOI:** 10.3390/ijerph191811679

**Published:** 2022-09-16

**Authors:** Shuai Li, Zeming Zhao, Boyi Hu, Tubing Yin, Gong Chen, Guohui Chen

**Affiliations:** 1 School of Resources and Safety Engineering, Central South University, Changsha 410083, China; 2 Hu Nan Shizhuyuan Non-Ferrous Metal Limited Liability Corporation, Chenzhou 423037, China

**Keywords:** open-pit mine, slope stability, numerical simulation, angle optimization

## Abstract

The stability of high and steep slopes in open-pit mines is closely related to the mine operations and the lives of the surrounding residents, so it is important to ensure the safety and stability of the slopes. Hazard classification and stability analysis of high and steep slopes under different working conditions are studied using the Shizhuyuan non-ferrous metal mine from underground to open-pit mining as a typical example. Firstly, data on rock mechanics parameters were obtained through site investigation and sampling. Then, the slope model of the open-pit mine was established and some slopes were selected in the model for qualitative and quantitative analysis. The strength reduction method and the limit equilibrium method were used to calculate the safety factor under each working condition and point out the potential instability areas. The results show that the selected slopes are safe and stable under all working conditions. Finally, on the premise of maintaining the safety and stability of the mine, the final slope angle was optimized from the original 45°21′35″ to 55°30′41″ to reduce production costs and increase mining efficiency. The final open-pit boundary that meets the stability requirements was eventually obtained.

## 1. Introduction

When high and steep slopes in mines are damaged, the surrounding environment and mine production can be greatly affected. For example, a major mining slope failure occurred in July 2012 on the east wall of the LAB Chrysotile mine in Canada. The major consequence of this failure was the loss of the local highway (Road 112), the main economic link between the region and the Northeast USA [1]. On 17 November 2016, an unforeseen large slope failure occurred in a copper open-pit mine, causing several casualties among the mine workers that were operating in the area [2]. A quarry failure along the slopes of the Wai Khar open-pit jade mine in Hpakant, Myanmar, led to the deaths of at least 172 jade miners on 2 July 2020 [3]. Therefore, it is essential to carry out safety assessments of the slopes of mines.

The limiting equilibrium method is the traditional method used to determine the stability of slopes. It has been applied and innovated by many scholars in a variety of cases, such as the reliability of four widely used limit-equilibrium-based methods, including simplified Bishop [4], simplified Janbu [5,6], Morgenstern–Price [7,8], and Spencer’s methods [9]. The jointly distributed random variables (JDRV) method is used as an analytical method to compare the reliability of four widely used limit equilibrium methods for slope stability analysis. Janbu and Bishop methods are those with upper and lower probabilities of failure, respectively, in two conditions with and without considering the cross-correlation between cohesion intercept (*c*) and angle of shearing resistance (*φ*) [10]. Numerical models for the present mining area and final mining area of the original scheme were established to analyse slope stability by the limiting equilibrium method [11]. The advent of computers allowed the use of the finite element method to produce results such as safety factors and deformation curves for slopes. Hong Zheng et al. (2006) demonstrated that while the finite element method is utilized for analysing slope stability, the critical slip lines based on the overloading definition might be for the most part shallower than those by the strength reserving definition and the conventional limit equilibrium methods, and hence the design based on the overloading definition might also be shallower [12]. In comparing various slope stability calculation methods, it can be found that the limit equilibrium method can program the calculation process of the slope, especially the search for the most dangerous sliding surface, which makes the complex calculation problem simple and fast. However, this method does not take into account the stress–strain relationship and the actual working conditions of the soil itself, but only uses artificial virtual states to find the safety factor. The advantage of the finite element method is that it partially takes into account the inhomogeneity and discontinuity of the rock mass of the slope and avoids the disadvantages of the limit equilibrium method, which is too simplified, but it is not ideal for solving problems such as large deformation, discontinuities in the rock mass, infinite domain, and stress concentration [13,14,15,16].

With the development of technology, techniques to study slope stability have gradually matured. The stability can be analysed using probabilistic methods. The deterministic approach was found to be inappropriate to analyse the stability of a slope having rock mass with variable properties [17]. The probabilistic approach offers a major advantage over the traditional deterministic method in that it accounts for the different degrees of variability and uncertainty often encountered in rock properties [18]. Meanwhile, it can be analysed by integrated remote sensing and GIS techniques. The susceptibility of slopes in open-pit coal mines to various modes of failure (i.e., plane, wedge, circular, and toppling failure) could be envisaged by virtue of processing and analysis of pertinent satellite data. All these data were subsequently used to create different thematic maps. Advanced analysis for extraction of lineament attributes was also undertaken [19]. In addition, the combination of kinematics and stereographic projection methods was used to analyse slope stability in open-pit mines. Cem Kincal (2014) performed a stability assessment of the already failed and standing sloped bench faces in the open-pit mine using stereographic projection techniques and software [20]. Shi-Gui Du et al. (2022) graded kinematic analysis using stereographic projection techniques. The stability assessment result showed good agreement with field observations, illustrating the ability of the proposed method to effectively predict the stability of open-pit mining slopes [21].

## 2. Engineering Background

The Hu Nan Shizhuyuan Non-ferrous Metal Limited Liability Corporation (hereinafter referred to as “Shizhuyuan Mine”) is located in Su Xian District, Chenzhou City, Hunan Province. The Shizhuyuan mine covers an area of 30.669 km^2^, with a production scale of 3.5 million *t*/*a* and a mining elevation of 0–1220 m. Five major deposits have been explored and developed, namely the Chaishan lead–zinc mine, the Chaishan molybdenum–bismuth–tungsten mine, the Jiweishan copper–polymetallic mine, the Shizhuyuan tungsten–polymetallic mine, and the Jiweishan lead–zinc mine.

**Figure 1:**
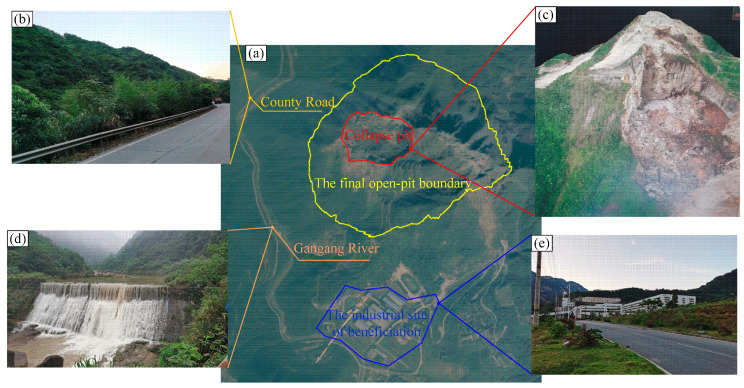
The open-pit mine and surroundings areas: (**a**) the satellite view of the surface of the open-pit mining area; (**b**) county road; (**c**) collapse pit model; (**d**) Gangang River; (**e**) the industrial site of beneficiation.

The Shizhuyuan mine is located about 26 km southeast of Chenzhou City, Hunan Province, with geographical coordinates of 113°07′30″ to 113°10′41″ east longitude and 25°42′13″ to 25°55′46″ north latitude. The mine is located in mountainous terrain with precipitous cliffs, rivers, and valleys, with a particularly dense distribution of high and steep slopes with slope angles above 60°, and the lower part of the slope is the Gangang River. The open-pit mine and surrounding areas are shown in Figure 1: (a) is the satellite view of the surface of the open-pit mining area; (b,d,e) are photographs taken on site; (c) shows a visual digital elevation model generated using UAV remote sensing technology to capture images and elevation data within the final open-pit boundary (including the collapse pit), which can be used for subsequent 3D modelling and analysis. The following facilities that exist on the surface near the final open-pit boundary require protection:

(1) The main surface river present in the vicinity of the mine site is the Gangang River, which flows in a south to north direction through the mine site from the western part of the mine site, with a low run-off and the main source of recharge being rainfall. Most of the ore bodies in the mine area are located above the local erosion datum.
(2) There is a county road from Chenzhou to Dongbo on the west bank of the Gangang River, which is a two-way dual carriageway passing through the mine site from north to south.
(3) The southern part of the final open-pit boundary is the industrial site of beneficiation, and there are factory buildings and other structures of the beneficiation plant.

With the rapid depletion of stockpiled ore and rich ore, the significant decline in the grade of raw ore, and the increasing cost of production, in order to maintain the economic efficiency and sustainable development of the enterprise, the mine currently adopts a fully underground mining method, based on the advantages of shallow burial and large reserves of ore bodies, the design of the open-pit mining method for ore bodies within a reasonable stripping ratio of the open-pit boundary, and the underground mining method for ore bodies outside the open-pit boundary. However, when open-pit mining is used, the safety and stability of the artificially high and steep slopes and the original natural slopes are closely related to the production safety of the mine, and once the open-pit slopes are destabilized, it may affect the production of the mine or cause a safety accident. Therefore, in order to investigate the impact of the slopes constructed in this open-pit mine on the surrounding environment and mining operations under different working conditions, the magnitude of the risk and safety stability needs to be evaluated [22,23].

**Figure 2:**
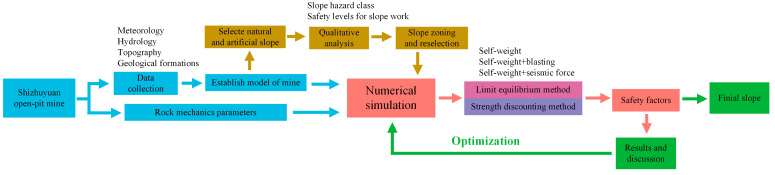
Flow diagram of stability analysis.

In order to systematically understand the slope stability of the Shizhuyuan mine, ensure the safety of the excavation process, and analyse the feasibility of its open-pit mining solution, this study comprehensively presents the following aspects and the flow diagram of stability analysis shown in Figure 2:

(1) The Shizhuyuan mine site was surveyed to collect complete information on meteorology and hydrology, topography, and geological formations along the project route. After samples were taken in the field, they were taken to the laboratory to determine the mechanical parameters. The parameters were reduced by Hoek–Brown strength criterion to obtain the final rock mass calculation parameters.
(2) The information collected was used to construct a slope model of the mine site, to qualitatively analyse the selected slopes to determine the damage pattern of the slopes, to alarm the zoning based on the results, and to select representative slopes in each zoning in preparation for the next step of numerical simulation.
(3) The factor of safety of the slope selected after the above zoning was calculated based on the strength reduction method, while its potential instability area was found based on the distribution of its plastic zone.
(4) Slide software was used to generate a number of folded sliding surfaces based on the potential instability areas of each slope, and then the factor of safety of these surfaces was calculated based on the simplified Bishop method, the simplified Jambu method, and the Morganstern–Price method of limit equilibrium theory, under the conditions of self-weight, self-weight + blasting, and self-weight + seismic force.
(5) The safety factor of the slope when increasing the slope angle was calculated and analysed, providing a basis for optimizing the step slope angle, the final slope angle, and the final open-pit boundary at the preliminary design stage.

### 2.1. Hydrogeological Conditions

The Shizhuyuan tungsten–polymetallic mine is located in the northern part of the middle section of the east–west tectonic zone of the Nanling, at the rising end of the Dongbo complex oblique between the Xishan backslope and the Wugai Mountain backslope. The stratigraphy in the area is strongly folded, with frequent magmatic activity and complex fracture structures. The mine is located on the northern side of the subtropical Nanling Mountain System, with obvious mountainous climate characteristics. The temperature is low throughout the year, the temperature difference between day and night and the four seasons varies greatly, and there is a lot of cloudy fog and abundant rainfall, with the rainy season mostly concentrated from April to June. According to the Chenzhou weather station, the annual rainfall ranges from 901.6 to 2247.6 mm, with a maximum daily rainfall of 139.9 mm.

The mine is an underground to open-pit mine, and the underground mining method is crumbling and the surface has formed a collapse pit. The collapse area and open pit formed by mining are conducive to the collection and infiltration of atmospheric rainfall, and therefore atmospheric rainfall is the most important factor in the water filling of the deposit. The ore body itself (skarn) and the surrounding rocks (marble and granite) are the main aquifer in the mine area and form the direct roof of the ore body, which is a direct factor in the water filling of the deposit, but the water richness of this aquifer is weak, and the water barrier of the granite and granite porphyry around the mine area and at depth is good, which has less influence on the water filling of the deposit. The main surface river in the vicinity of the mine site is the Gangang River, but the river has minimal impact on the mining of the ore body. The majority of the ore body is located above the local erosion datum, which provides favourable conditions for the self-flow drainage of pit water.

In summary, the mine area has poor groundwater recharge conditions and simple hydrogeological boundaries, while all pioneering works and mining operations during open-pit mining are located above the lowest groundwater erosion datum. Therefore, the stability analysis of the high and steep slopes of open-pit mining under normal working conditions does not consider the role of groundwater for the time being.

### 2.2. Mechanical Parameters of Rock Mass

**Figure 3:**
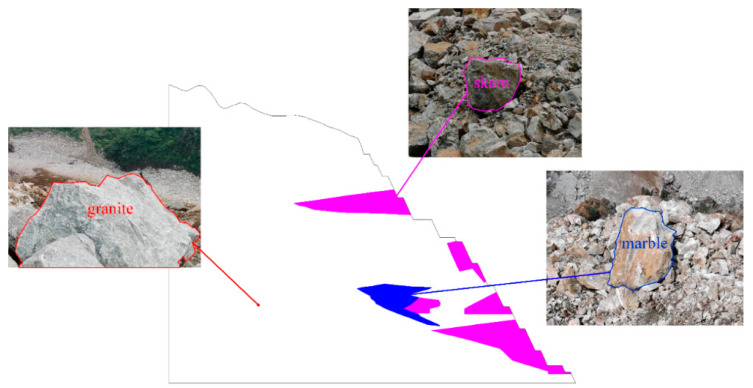
The geological section of the R1 slope of the open pit.

After on-site rock sampling and laboratory rock mechanics tests, the mechanical parameters of the rock were obtained, but they could not be directly used for theoretical calculations. This is because the rock mass mainly consists of rock and structural faces, and the mechanical parameters of the rock mass are significantly different from those of the intact rock mass under the weakening effect of the cutting of the structural faces, so the mechanical parameters of the rock need to be reduced. Therefore, the mechanical parameters of the rock were reduced to rock mass mechanics based on Hoek–Brown strength criterion. The final obtained mechanical parameters of the slope rock masses are shown in Table 1. Among them, the mechanical parameters of the skarn rock mass are all used for the ore body, and the surrounding rocks are divided into two types: granite and marble. For example, the geological section of the R1 slope of the open pit is shown in Figure 3.

## 3. Slope Hazard Classification

Before the calculation of safety factors for high and steep slopes in the mining area, a qualitative analysis of the selected slopes should be carried out based on the results of the site survey and relevant experience, including the classification of stability zones and potential damage modes of the slopes. A representative set of slopes is then selected through the above analysis and a reasonable method is used to carry out the slope stability calculation and analysis.

In stability zoning, engineering geological zoning is carried out first, dividing the slopes according to the principle that the rock properties, formations, and engineering geological conditions are the same or consistent. In the same geological zone, the geometrical elements of the slope and the slope section are basically the same and can be characterized by the same section and the same calculation parameters.

Open-pit slopes shall be classified according to their final height into four classes:

(1) Ultra-high: *H* greater than 500 m;
(2) High: *H* greater than 300 m less than or equal to 500 m;
(3) Medium: *H* greater than 100 m less than or equal to 300 m;
(4) Low: *H* less than or equal to 100 m.

The classification of slope hazards is shown in Table 2. The classification of safety levels of slope engineering is shown in Table 3 [24] (pp. 6–7).

Based on the above criteria, firstly, according to the engineering geological zoning requirements, the designed slopes of the final boundary of open-pit mining are divided into two categories: natural slopes refer to the original slopes of the mountain that may be affected by open-pit mining, while artificial mining slopes refer to the unnatural slopes formed by open-pit mining to the final boundary, and each of these two categories of slopes has similar engineering conditions.

**Figure 4:**
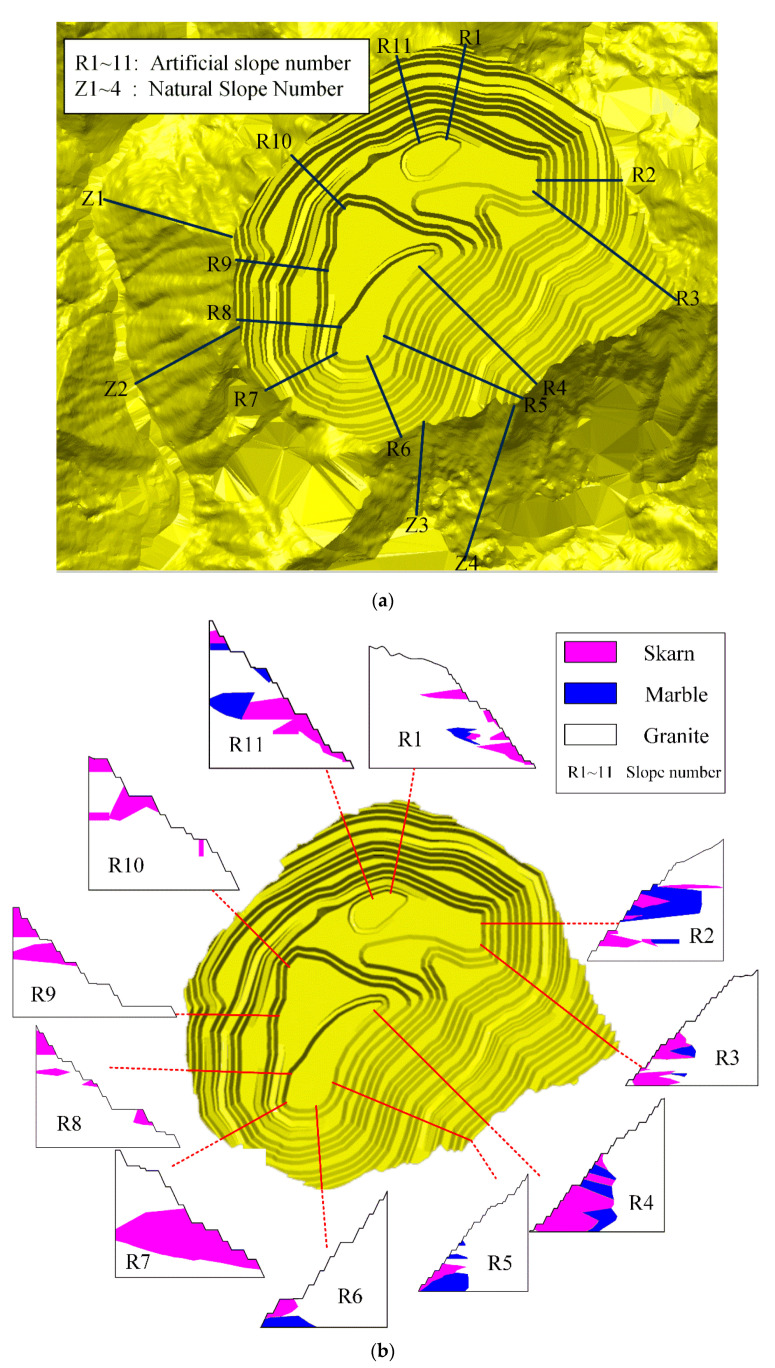

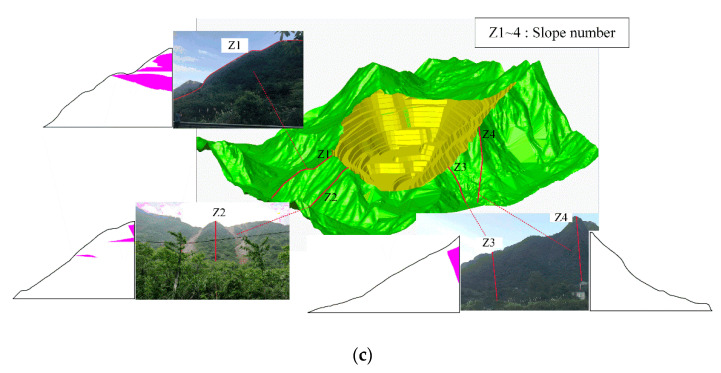
The slope section models: (**a**) diagram for the location of the selected slopes in the mining area; (**b**) artificial mining slopes; (**c**) natural slopes.

Then, in the same engineering geological subzone, it is divided into different sections with basically the same geometrical elements and slope section, and the same calculation parameters can be used. Each section then selects a typical location of the section to characterize, thus determining the artificial mining slope sections for 11 slopes numbered R1~R11, and the natural slope for four slopes numbered Z1~Z4. The digital model of the completed open-pit slope and the location of the selected slopes in the mining area are shown in Figure 4. The digital elevation model was generated by combining the elevation information obtained from the above-mentioned UAV remote sensing technology with the plan for open-pit mining in the software.

Although the surface river, the road, and the industrial site of beneficiation are all 300 m away from the final boundary, the slopes of the open-pit mine are classified as Class II or Class III according to the potential hazard posed by each slope, taking into account the potential impact of the slopes on production activities within the boundary and on the surface protection facilities outside the boundary. The results of the classification of the height class, hazard class, and engineering safety class of each side slope are shown in Table 4.

**Figure 5:**
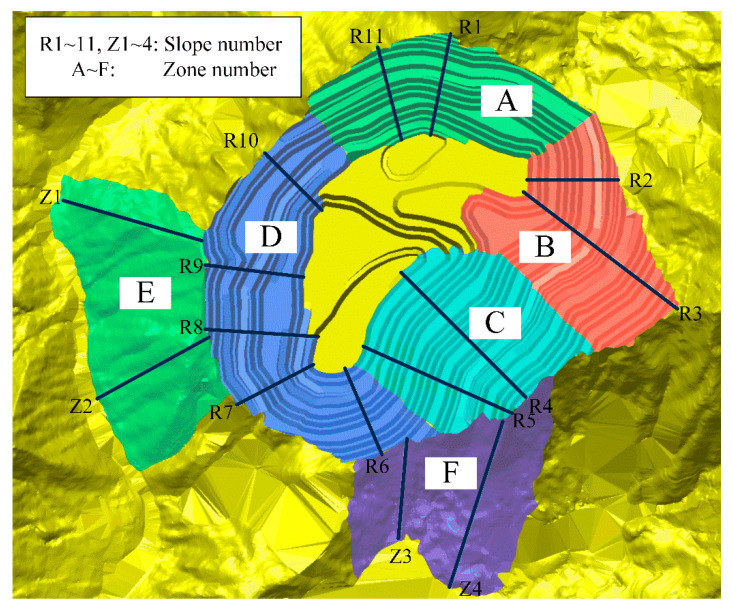
Diagram for mining slope zoning.

According to the classification of slope grades in the table, the geometric parameters of the selected slopes, engineering geological conditions, and other factors, the artificial mining slopes in the high and steep slopes are divided into four zones, A, B, C, and D (A: R1, R11; B: R2, R3; C: R4, R5; D: R6~R10), and the natural slopes are divided into two zones, E and F (E: Z1, Z2; F: Z3, Z4). The zoning diagram is shown in Figure 5.

As the slopes in each of the zones divided are homogeneous and similar, one slope in each group can be selected separately as a representative for the study. Through exchange and discussion and comprehensive analysis, R1, R3, R4, R8, Z1, and Z4 were selected as representatives of each group of slopes, respectively. It can be found that the height of the selected slope is higher in the same group, which means that the possibility of occurrence of geological disasters such as landslide, rock fall, and collapse is higher and the potential danger is greater, which will cause more serious potential economic losses and casualties, so its risk level is mostly identified as II. Safety levels of slope engineering are I and II, and only R8 has a Class III because its slope height is only 231 m.

## 4. Numerical Simulation

Numerical simulation of slopes in open-pit mines uses both the strength reduction method and the limit equilibrium method together. The strength reduction method was first used by Zink Iewic in 1975 for slope stability analysis. Due to poor calculation conditions at that time, this method was rarely used, but with the rapid development of information technology, the strength reduction method slowly became popular until now it has become the main method for slope stability calculation. The traditional calculation method for slope stability analysis is the limit equilibrium method, which is widely used in engineering. This method divides the landslide body into a number of strips, establishes the equilibrium equation for the forces acting on these strips, and solves for the safety factor. There are many methods based on this principle, such as the Fellenius method, Bishop method, Janbu method, Dn method, Sarma method, Spencer method, and Morgenstern–Price method.

### 4.1. Geometric Modelling

When building the numerical model in the software, the Mohr–Coulomb model is chosen, a theory that provides a more comprehensive response to the strength characteristics of rocks and soils and is applicable to both brittle and plastic materials [25]. The numerical analysis method employed is the three-dimensional fast Lagrangian method, which simulates the three-dimensional mechanical behaviour of geotechnical soils. The method involves dividing the computational region into a number of tetrahedral cells, each of which follows a specified linear or non-linear intrinsic structure relation for a given boundary condition, and the cell mesh can be deformed as the material deforms if the cell stresses make the material yield or produce plastic flow. This algorithm is ideally suited to modelling large deformation problems.

Firstly, a model of the above selected representative slope is chosen in FLAC^3D^, the strength reduction method is used to calculate the safety and stability coefficients, and the potential instability zones are identified according to the force cloud diagram in preparation for the next step of the limit equilibrium analysis [26]. Then, the geological conditions of the mining slopes are examined to identify their slope failure modes, analyse and specify their potential instability zones, specify their damage zones according to the different failure modes, calculate the safety stability coefficients using the limit equilibrium method, and determine the safety of the open-pit slopes.

According to the geological classification criteria for open-pit slopes in the Technical Code for Non-Coal Open-Pit Mine Slope Engineering (as shown in Table 5), the damage pattern of open-pit slopes can be determined [24] (p. 52).

As the underground to open-pit mining of the Shizhuyuan polymetallic mine is still in the feasibility study stage, the slopes within the open-pit boundary have not yet been actually formed, so with reference to geological data and combined with the results of the site investigation, the rock structure type of the slopes of the Shizhuyuan polymetallic mine open-pit mine belongs to the laminated structure, the shape of the structure body is mainly laminated and slab-like, the structure surface has laminae, lamellae, and joints, and there are often inter-layer misalignments. Its deformation and strength characteristics are controlled by the combination of layers and rock strata and can be regarded as an elastic–plastic body, which may produce sliding, bending, and tensioning damage to rock strata and plastic deformation of weak rock strata. The degree of rock integrity of the slopes of open-pit mines is relatively intact. According to the analysis in Table 4, the slopes of the Shizhuyuan polymetallic mine open-pit mine are layered rock slopes, which are classified as the same tendency and oblique slopes, so the slope damage modes are planar, folded line, and wedge [27].

With the above simulation results, the area slope instability can be specified in the simulation software and the slope damage model of the open pit can be determined. Using this condition, the next step of the analysis is to artificially specify the damage range and path in the slope model, and to calculate the safety coefficients of the slope instability area under three different operating conditions, self-weight, self-weight + blasting, and self-weight + seismic force, respectively, using the limit equilibrium method. After deriving the safety factor of the open slope of the Shizhuyuan mine, it is necessary to analyse its stability, explore whether the angle of the slope construction is reasonable, and give optimization according to the current engineering status and geological conditions.

### 4.2. Results and Analysis

#### 4.2.1. Results of Slope Simulation

(1) Strength reduction method

**Figure 6:**
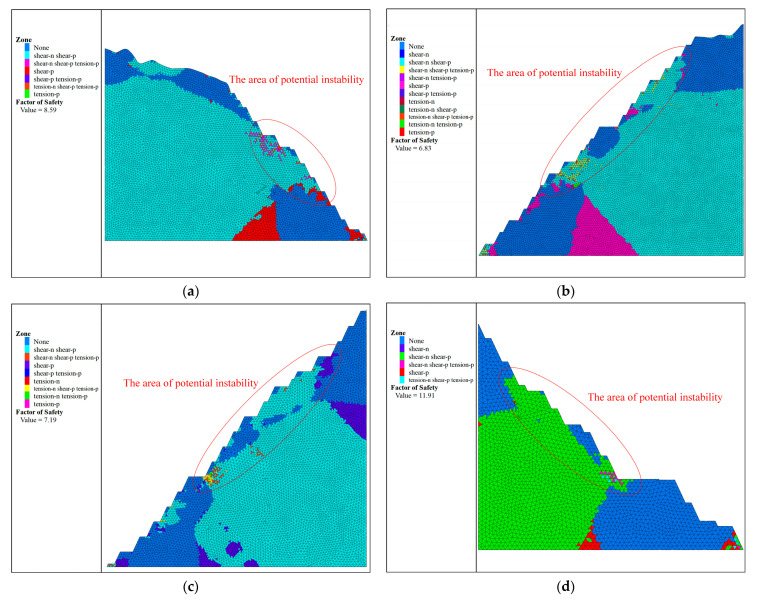

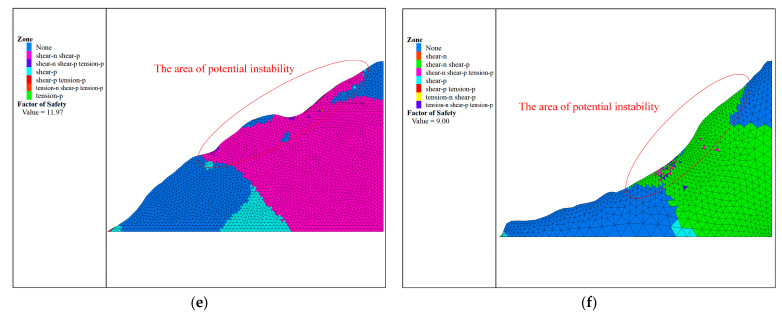
Calculation results of the strength reduction method: (**a**) R1; (**b**) R3; (**c**) R4; (**d**) R8; (**e**) Z1; (**f**) Z4.

After each 2D section model is stretched 20 m along the vertical direction, the FLAC^3D^ calculation model of the 2D slope is obtained, and the strength reduction method of FLAC^3D^ is used to calculate and analyse it under the normal working conditions of self-weight stress. The calculation results are shown in Figure 6, which shows the plastic zone condition of each refuge break in addition to the safety factor and the potential instability area.

The safety factor criteria required for different slope engineering safety levels are different, and the specific requirements for design safety factors under self-weight conditions are 1.25~1.20 for Class I safety level, 1.20~1.15 for Class II, and 1.15~1.10 for Class III. The calculation results of slope safety factors for the strength reduction method are shown in Table 6.

From the results obtained above, it can be concluded that the slopes constructed in this open pit are safe and stable, with all safety factors higher than the safety factor criteria of the slope works. Among them, the safety factor of R3 (6.83) is the smallest among the artificial mining slopes and the safety factor of Z4 (9.00) is the smallest among the natural slopes. These two slopes should be the focus of future work to ensure that the relevant works are carried out successfully.

(2) Limit equilibrium method

After calculation by the strength reduction method, the resulting slope instability area can be subjected to a limit equilibrium analysis to plan its damage extent according to the failure model. The stability coefficients of the slope are then calculated for each of the three working conditions (self-weight, self-weight + blasting, self-weight + seismic force) using the three calculation methods (Bishop, Janbu, and Morgenstern–Price).

According to the Seismic Ground Motion Parameters Zonation Map of China, the peak acceleration of ground shaking at the mine site is 0.05 *g* and the characteristic period of the response spectrum is 0.35 s [28] (pp. 128–140). The comparison table between the peak ground motion acceleration and the seismic intensity of the site is shown in Table 7, and the seismic intensity is VI. Referring to the Code for Seismic Design of Buildings, the seismic impact coefficient of the building structure shall be determined according to the intensity, site category, design seismic grouping, and self-oscillation period of the structure, as well as the damping ratio. The maximum value of its horizontal seismic impact coefficient should be adopted according to Table 8 [29] (pp. 31–43). The seismic intensity of the Shizhuyuan mine site is 6 degrees, the horizontal seismic impact factor is taken to be 0.04, and the vertical seismic impact factor is taken to be 65% of the former at 0.026, and these are taken as the seismic parameters for the seismic condition. In addition, a horizontal seismic impact factor of 0.0381 was taken as the blast impact condition when only the self-weight + blasting condition was considered [30].

**Figure 7:**
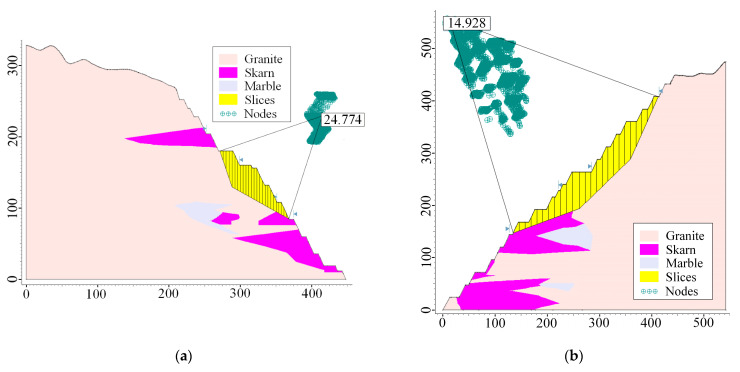

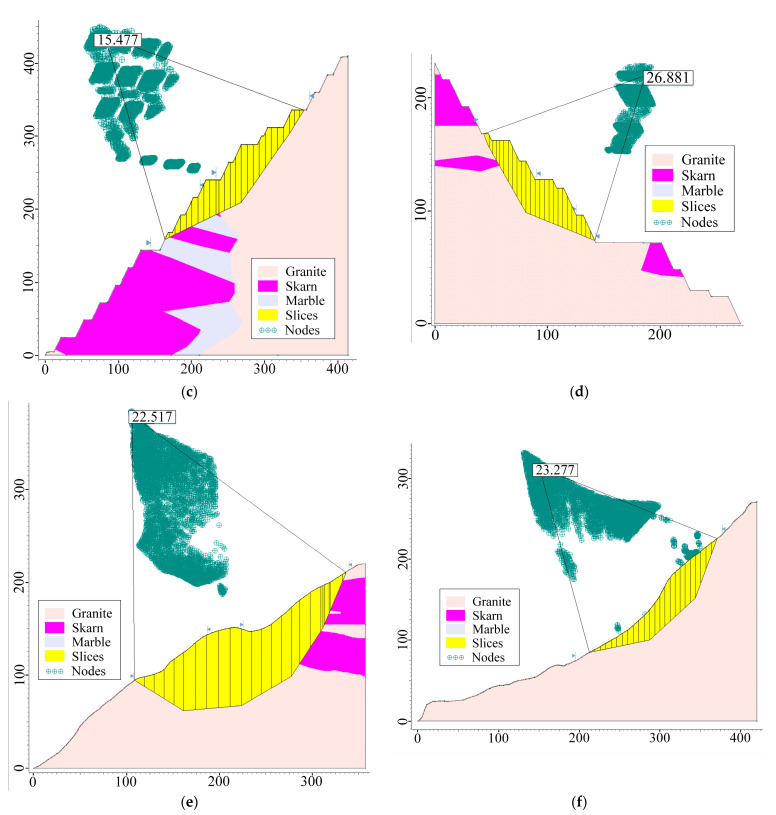
Calculation results of the limit equilibrium method (self-weight): (**a**) R1; (**b**) R3; (**c**) R4; (**d**) R8; (**e**) Z1; (**f**) Z4.

**Figure 8:**
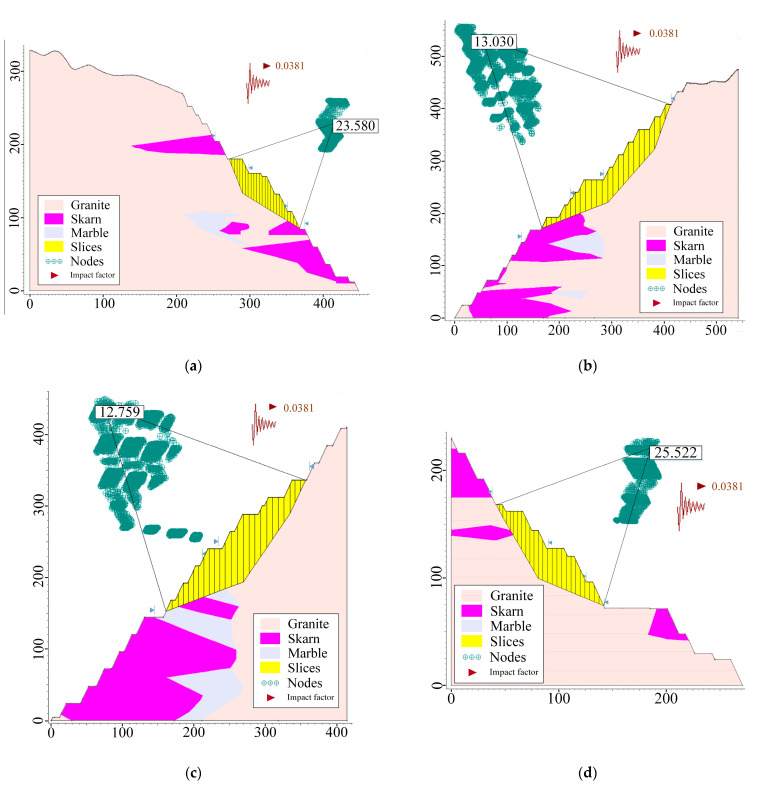

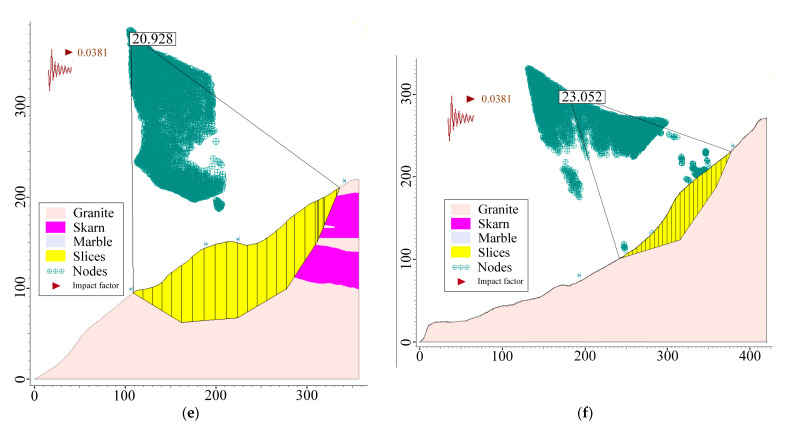
Calculation results of the limit equilibrium method (self-weight + blasting): (**a**) R1; (**b**) R3; (**c**) R4; (**d**) R8; (**e**) Z1; (**f**) Z4.

**Figure 9:**
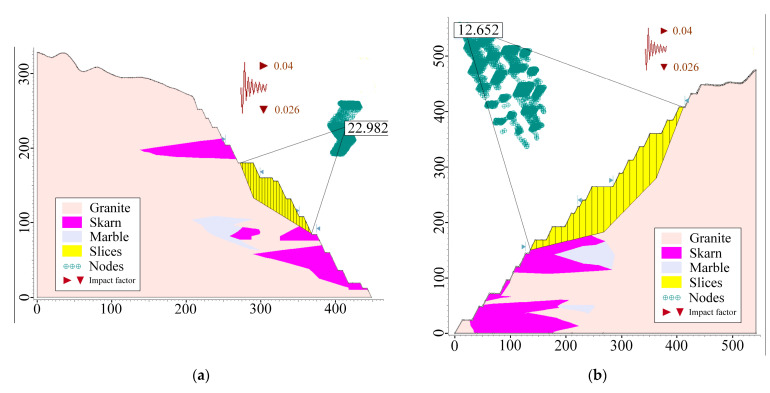

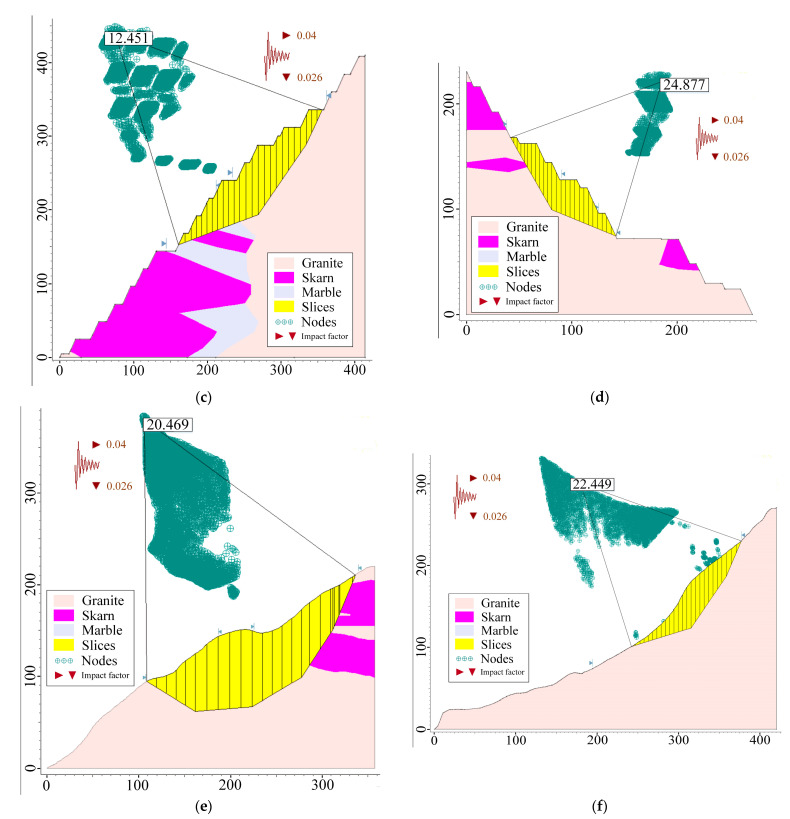
Calculation results of the limit equilibrium method (self-weight + seismic force): (**a**) R1; (**b**) R3; (**c**) R4; (**d**) R8; (**e**) Z1; (**f**) Z4.

Finally, compared to other methods, the Morgenstern–Price method is more advanced and efficient, and the image display is more beautiful and straightforward, so it is used as an example [31,32]. The results for the self-weight condition are shown in Figure 7, and the results for self-weight + blasting and self-weight + seismic force are shown in Figure 8 and Figure 9, respectively. The calculation results of slope safety factors for the limit equilibrium method are shown in Table 9.

Generally, by observing the results of the safety factors, it is found that the safety factor of the slope is higher than the slope design safety factor criteria in all conditions. On average, the smallest safety factor of the artificial mining slopes is R3 and the smaller safety factor among the natural slopes is Z4. Therefore, it can be concluded that the open-pit slopes in the final boundary of Shizhuyuan are safe and stable.

Furthermore, comparing the safety factors for the three different working conditions under the same calculation method, it can be found that the pattern of the safety factors is self-weight working condition > self-weight + blasting > self-weight + seismic force. The percentage effects of blasting and seismic forces on the safety factor are shown in Table 10. It can be seen from the effects of blasting and seismic forces on the safety factors that seismic force and blasting have a certain influence on the safety and stability of slopes, but the factor does not change significantly, and its absolute value is generally less than 10%. The difference between the results for the self-weight condition and those for the self-weight + seismic force condition is very small, indicating that blasting activities and general seismic activities have a small influence on the slopes of an open-pit mine and do not negate the conclusion that the slopes are safe and stable.

#### 4.2.2. Optimization of Stope Slope Angle

By analysing the above calculation results, it is found that the safety factor under each working condition using various methods is higher than the safety factor criteria of the slope works, which indicates that there is still room for optimization of the existing engineering design and the slope angle of the slope is on the small side. Therefore, the R3 slope (448 m) with lower safety stability can be selected as the object of analysis in the above analysis, and its final slope angle can be increased for stability analysis to ensure a more reasonable final slope angle under the premise of overall safety and stability of the slope, thus reducing the project production cost.

Referring to the reference table for step slope angles in the Mining Design Manual—Deposit Mining Volume (Table 11), the adjustment of the final side slope angle is within a reasonable range. The main lithology of the R3 side slope is granite, which has a rock hardness coefficient of 15 < *f* < 20 according to the results of the laboratory rock mechanics tests, so it is recommended that the step slope angle be optimized to 80° [33,34].

**Figure 10:**
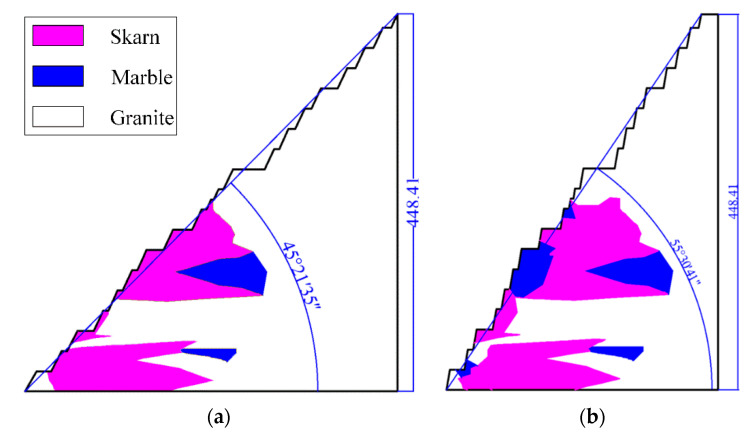
R3 slope profile after step slope angle adjustment: (**a**) pre-adjustment; (**b**) after adjustment.

**Figure 11:**
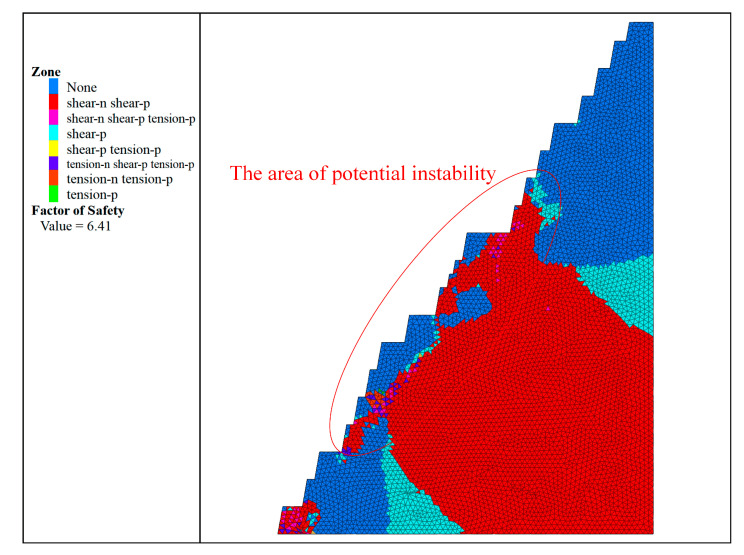
Results of the adjusted R3 slope for the strength reduction method.

**Figure 12:**
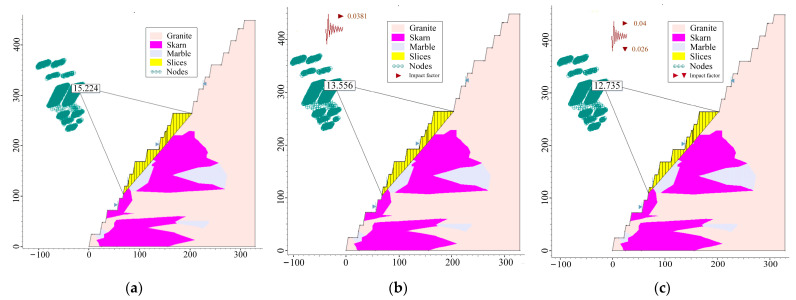
Results of the adjusted R3 slope for the limit equilibrium method: (**a**) self-weight; (**b**) self-weight + blasting; (**c**) self-weight + seismic force.

Therefore, the step slope angle of the R3 slope section is changed to 80°, and the widths of its safety platform, sweeping platform, and transport platform are kept unchanged. After adjustment, a two-dimensional slope section with a higher final slope angle was obtained, and the comparison diagram with the original section is shown in Figure 10. The final slope angle is 55°30′41″. After carrying out the same analysis process as above for the adjusted R3 slope, the results of its strength reduction method were obtained as shown in Figure 11, the results of the limit equilibrium method were obtained as shown in Figure 12, and the results of the comparison between the adjusted and pre-adjusted slopes are shown in Table 12.

From the above table, it can be seen that the safety factor of the R3 slope after adjusting the step slope angle is greater than the design safety factor under various working conditions, and we also obtained the same result for the other slopes in the same calculation, which shows that the adjusted slope still meets the design requirements. In other words, the safety factor of the R3 slope is greater than the design safety factor when the step slope angle is increased from 65° to 80° and the final slope angle is expanded from 45°21′35″ to 55°30′41″.

## 5. Discussion

This study is based on UAV remote sensing technology to create a visual elevation model for open-pit mining. The strength reduction method was used to find the potential instability areas and combined with the limit equilibrium method to calculate the safety factors of the selected slopes under each working condition. The slopes selected include both artificial and natural slopes within the final open-pit boundary, so the results of the study are more comprehensive and reliable.

After adjusting the step slope angle of the R3 slope with the minimum safety factor from 65° to 80°, the final slope angle increases from 45°21′35″ to 55°30′41″ and its calculation is also safe. However, considering the complexity of slope engineering problems and ensuring the safety and stability of the slopes, it is recommended that the step slope angle should not be too large, the optimized value range for the step slope angle is 70°~75°, and the final slope angle is 45°~50°. Therefore, an optimized slope angle can significantly reduce the stripping ratio and construction costs, while ensuring safety.

This study is based on the existing open-pit mining design. If a larger final slope angle is to be adopted, a comprehensive and meticulous analysis of the new final open-pit boundary is required. One important future direction of optimizing slope angles is the consideration of weathering in addition to the hardness coefficient. Thus, future research may still need to be improved and refined.

## 6. Conclusions

In this study, slope failure in the Shizhuyuan polymetallic open-pit mine was studied in detail. It mainly included geological settings, failure characteristics, failure model, relevant numerical simulation, and slope angle optimization. The following conclusions can be drawn:

(1) Through site investigation and research, the slopes of the Shizhuyuan polymetallic mine open-pit mine are hazard Class II and III, and safety levels of slope engineering are mostly Class I and III. In addition, the slopes of the mine are laminated rock slopes, which are classified as the same tendency and oblique slopes, so the slope damage modes are planar, folded line, and wedge.
(2) The smallest factor of safety for the R3 slope was calculated using FLAC^3D^ software, with 6.83 being larger than the safety factor criterion (1.25). The smallest safety factor calculated in Slide software for the open-pit slope under the three conditions of self-weight, self-weight + blasting, and self-weight + seismic forces is R3 for the Bishop method, with 10.954, 10.396, and 10.126 respectively, which are all larger than the criterion (1.25), and the absolute value of the effect of blasting and seismic forces is generally less than 10%. The results of the two software calculations are consistent, indicating that the slopes of the open pit are safe and stable and can meet the daily construction requirements such as rock drilling and blasting.
(3) After deriving the safety factors for each side slope, it was found that the values were higher than the safety criteria, so the angle of the mine side slope construction could be optimized. The step slope angle was increased from 65° to 80° and the final slope angle was expanded from 45°21′35″ to 55°30′41″. The safety factor of the improved slope was still calculated to be in line with the relevant safety standards, which provides a basis for optimizing the step slope angle, the final slope angle, and the final open-pit boundary at the preliminary design stage.

## Figures and Tables

**Table 1:** Rock mechanics parameters of slope rock after engineering reduction.

Rock Mass Types	Lithology	Elasticity Modulus (GPa)	Compressive Strength (MPa)	Strength of Extension (MPa)	Poisson’s Ratio	Unit Weight (kN·m^−3^)	Cohesive Force (MPa)	Internal Friction Angle (°)
Ore body	Skarn	45.420	108.050	9.76	0.2238	31.430	8.25	44.770
Surrounding rock	Marble	28.680	72.450	3.56	0.2680	27.700	3.07	40.150
Surrounding rock	Granite	37.670	102.590	7.04	0.2381	25.810	6.73	43.630

**Table 2:** Slope hazard class.

Slope Hazard Class		I	II	III
Potential for casualties		Possible	Possible	Impossible
Potential economic loss	Direct	≥1 million	0.5 million~1 million	≤0.5 million
	Indirect	≥10 million	5 million~10 million	≤5 million
Overall rating		Very serious	Serious	Not serious

**Table 3:** Classification of safety levels.

Safety Levels of Slope Engineering	Slope Height *H* (m)	Slope Hazard Class
I	*H* > 500	I, II, III
	300 < *H* ≤ 500	I, II
	100 < *H* ≤ 300	I
II	300 < *H* ≤500	III
	100 < *H* ≤300	II, III
	*H* ≤ 100	I
III	100 < *H* ≤ 300	III
	*H* ≤ 100	II, III

**Table 4:** Classification of the slopes.

Slope Zone	Number	Slope Height *H* (m)	Slope Height Classes	Hazard Classes	Safety Levels of Slope Engineering
Artificial mining slopes	R1	328	High	II	I
	R2	290	Medium	III	III
	R3	448	High	II	I
	R4	410	High	II	I
	R5	407	High	II	I
	R6	231	Medium	III	III
	R7	168	Medium	III	III
	R8	231	Medium	III	III
	R9	240	Medium	III	III
	R10	171	Medium	III	III
	R11	228	Medium	III	III
Natural slopes	Z1	220	Medium	II	II
	Z2	187	Medium	II	II
	Z3	120	Medium	III	III
	Z4	271	Medium	II	II

**Table 5:** Geological classification of slopes in open-pit mine.

Type of Slope Geological Structure		Characteristic	Failure Mode of Slope
Massive rock slope		The rock is essentially homogeneous, *D*_50_/*L*_c_ ≥ 0.02	Planar Wedge Toppling
Layered rock slope	The same tendency	*α* ≤ 30°; Level friction angle < *β* ≤ slope angle	Planar Folded line
	Oblique	30° < *α* ≤ 75°; level friction angle < combined screen intersection inclination ≤ slope angle	Wedge
	Others	Structural surface combinations do not directly control slope failure	Circular Composite
Cataclastic rock slope		Laminated or fragmented rock, D50/*L*_c_ < 0.02	Circular Composite
Granular medium slope		Strongly fractured, strongly weathered rock masses, weakly altered rock masses, all types of soils	Circular Composite

**Table 6:** Calculation results of the strength reduction method.

Slope Zone	Number	Safety Factor	Safety Levels for Slope Work	Safety Factor Criteria
Artificial mining slopes	R1	8.59	I	1.25~1.20
	R3	6.83	I	1.25~1.20
	R4	7.19	I	1.25~1.20
	R8	11.91	III	1.15~1.10
Natural slopes	Z1	11.97	II	1.20~1.15
	Z4	9.00	II	1.20~1.15

**Table 7:** The comparison table between the peak acceleration and the seismic intensity.

Peak Ground Motion Acceleration of Class II Site	0.04 *g* ≤ *a*_maxII_ < 0.09 *g*	0.09 *g* ≤ *a*_maxII_ < 0.19 *g*	0.19 *g* ≤ *a*_maxII_ < 0.38 *g*	0.38 *g* ≤ *a*_maxII_ < 0.75 *g*	*a*_maxII_ ≥ 0.75 *g*
Seismic intensity	VI	VII	VIIII	IX	≥X

**Table 8:** The maximum value of horizontal seismic impact coefficient.

Extent of Earthquake Impact	6 Degrees	7 Degrees	8 Degrees	9 Degrees
Frequently	0.04	0.08	0.16	0.32
Rarely	0.28	0.50	0.90	1.40

**Table 9:** Calculation results of limit equilibrium method.

Slope Zone	Number	Safety Factor								
		Self-Weight			Self-Weight + Blasting			Self-Weight + Seismic Force		
		Bishop	Janbu	M-P	Bishop	Janbu	M-P	Bishop	Janbu	M-P
Artificial mining slopes	R1	23.887	25.073	24.774	22.933	23.999	23.580	22.357	23.389	22.982
	R3	10.954	13.251	14.928	10.396	12.608	13.030	10.126	12.277	12.652
	R4	11.808	13.394	15.477	11.306	12.444	12.759	11.034	12.141	12.451
	R8	24.007	25.562	26.881	23.004	24.466	25.522	22.426	23.846	24.877
Natural slopes	Z1	22.209	22.144	22.517	20.615	20.559	20.928	20.121	20.056	20.469
	Z4	18.051	20.576	23.277	17.327	19.761	23.052	16.901	19.264	22.449

**Table 10:** Percentage effect of blasting and seismic force on safety factor.

Slope Zone	Number	Percentage Effect of Blasting and Seismic Force on Safety Factor					
		Bishop		Janbu		M-P	
		Self-Weight + Blasting	Self-Weight + Seismic Force	Self-Weight + Blasting	Self-Weight + Seismic Force	Self-Weight + Blasting	Self-Weight + Seismic Force
Artificial mining slopes	R1	−3.99%	−6.41%	−4.28%	−6.72%	−4.82%	−7.23%
	R3	−5.09%	−7.56%	−4.85%	−7.35%	−12.71%	−15.25%
	R4	−4.25%	−6.55%	−7.09%	−9.35%	−17.56%	−19.55%
	R8	−4.18%	−6.59%	−4.29%	−6.71%	−5.06%	−7.46%
Natural slopes	Z1	−7.18%	−9.40%	−7.16%	−9.43%	−7.06%	−9.10%
	Z4	−4.01%	−6.37%	−3.96%	−6.38%	−0.97%	−3.56%

**Table 11:** Step slope angle reference.

Items	Contents			
Rock hardness coefficient (*f*)	15~20	8~14	3~7	1~2
Step slope angle	75°~85°	70°~75°	60°~65°	45°~60°

**Table 12:** Summary of the results of the adjusted R3 slope stability analysis.

Type			Value	Safety Levels for Slope Work	Safety Factor Criteria
Step slope angle	Pre-adjustment		65°	—	—
	After adjustment		80°		
Final slope angle	Pre-adjustment		45°21′35″		
	After adjustment		55°30′41″		
Working conditions	Self-weight	Slide	15.224	I	1.25~1.20
		FLAC^3D^	6.41		
	Self-weight + blasting		13.556		1.23~1.18
	Self-weight + seismic force		12.735		1.20~1.15

## Data Availability

The data presented in this study are available on request from the corresponding author.
